# The Induction of Recombinant Protein Bodies in Different Subcellular Compartments Reveals a Cryptic Plastid-Targeting Signal in the 27-kDa γ-Zein Sequence

**DOI:** 10.3389/fbioe.2014.00067

**Published:** 2014-12-11

**Authors:** Anna Hofbauer, Jenny Peters, Elsa Arcalis, Thomas Rademacher, Johannes Lampel, François Eudes, Alessandro Vitale, Eva Stoger

**Affiliations:** ^1^Department of Applied Genetics and Cell Biology, University of Natural Resources and Life Sciences, Vienna, Austria; ^2^Institute of Molecular Biotechnology, RWTH Aachen University, Aachen, Germany; ^3^Agriculture and Agri-Food Canada, Lethbridge, AB, Canada; ^4^Institute of Agricultural Biology and Biotechnology, National Research Council (CNR), Milan, Italy

**Keywords:** protein bodies, molecular farming, subcellular targeting, intermembrane space, plastid import, recombinant protein

## Abstract

Naturally occurring storage proteins such as zeins are used as fusion partners for recombinant proteins because they induce the formation of ectopic storage organelles known as protein bodies (PBs) where the proteins are stabilized by intermolecular interactions and the formation of disulfide bonds. Endogenous PBs are derived from the endoplasmic reticulum (ER). Here, we have used different targeting sequences to determine whether ectopic PBs composed of the N-terminal portion of mature 27 kDa γ-zein added to a fluorescent protein could be induced to form elsewhere in the cell. The addition of a transit peptide for targeting to plastids causes PB formation in the stroma, whereas in the absence of any added targeting sequence PBs were typically associated with the plastid envelope, revealing the presence of a cryptic plastid-targeting signal within the γ-zein cysteine-rich domain. The subcellular localization of the PBs influences their morphology and the solubility of the stored recombinant fusion protein. Our results indicate that the biogenesis and budding of PBs does not require ER-specific factors and therefore, confirm that γ-zein is a versatile fusion partner for recombinant proteins offering unique opportunities for the accumulation and bioencapsulation of recombinant proteins in different subcellular compartments.

## Introduction

Plants are versatile platforms for the production of recombinant pharmaceutical proteins and peptides because they are safe, scalable, and allow long-term protein storage (Ma et al., 2013; Rybicki et al., 2013; Stoger et al., 2014). It is usually advantageous if the protein accumulates at a high concentration and remains stable after harvest, particularly in the case of antigens, antibodies, and enzymes produced for oral application, where doses must be up to 1000-fold higher than parental formulations to ensure that sufficient amounts of the protein survive proteolytic digestion.

The stability of recombinant proteins upon administration can be increased by *in vitro* encapsulation, which usually involves spray or freeze drying methods and mixing with plant-derived components such as cereal storage proteins (Zhong and Jin, 2009; Zou and Gu, 2013). Prolamin-type storage proteins (e.g., maize zeins) are often used for this purpose because their film-building properties allow them to form coatings and microparticle formulations, and they also have GRAS food use status (generally recognized as safe by the U.S. Food and Drug Administration) and high resistance to digestion (Liu et al., 2005; Wang et al., 2011; Lau et al., 2013). When using plants as production hosts, it is therefore appealing to attempt microencapsulation *in vivo* by directly incorporating the recombinant protein into protein storage bodies (Hofbauer and Stoger, 2013). This is the typical strategy used with seed-based production systems, where the recombinant protein is often targeted so that it accumulates in prolamin-containing storage organelles. Several studies have shown that recombinant proteins incorporated in storage organelles are protected from proteolytic digestion in simulated gastric fluids and are more effective following oral delivery, indicating the potential of this strategy for the bioencapsulation of recombinant pharmaceutical proteins and their efficient mucosal delivery (Chikwamba et al., 2003; Nochi et al., 2007; Takagi et al., 2010; Wakasa et al., 2013).

Protein bodies (PBs) form naturally in the endoplasmic reticulum (ER) of developing cereal endosperm cells, but the expression of recombinant proteins fused to assembly sequences can induce analogous organelles in tissues such as leaves, which usually lack these structures. Sequences that can induce the formation of ectopic PBs and thus increase the yield and stability of recombinant proteins include those derived from cereal prolamins, synthetic elastin-like peptides, and fungal hydrophobins (Floss et al., 2008; Conley et al., 2009; Torrent et al., 2009b; Gutierrez et al., 2013; Shigemitsu et al., 2013).

One of the most widely used assembly sequences is found near the N-terminus of the 27 kDa γ-zein, a member of the major prolamin-type storage protein family in maize (Shewry and Halford, 2002). About 15 zein genes (divided into α-, β, δ, and γ-zeins) are expressed at high levels in maize endosperm and condense together in the ER, to from the heteropolymeric PBs that often bud off the ER as distinct round shaped structures that maintain polyribosomes on the cytosolic surface (Lending and Larkins, 1989). The γ-zeins form the peripheral layer of PBs and play a structural role in their biogenesis (Ludevid et al., 1984). Ectopically expressed zein under the control of the constitutive 35S promoter forms ER-resident PBs in vegetative tissues, underscoring its intrinsic compartment-forming properties in the absence of tissue-specific factors (Bagga et al., 1995). The PB-inducing properties of 27 kDa γ-zein have been causally linked to its N-terminal half, comprising a domain with two Cys residues that follows the signal peptide, a repeated Pro-rich domain forming an amphipathic helix, and a third domain that includes four additional Cys residues (Geli et al., 1994). Mutagenesis of the Cys residues or the amphipathic repeat in fusion proteins or full length 27 kDa γ-zein indicate that the repeat favors PB formation, but the Cys residues have a fundamental role (Mainieri et al., 2004; Llop-Tous et al., 2010). Several reports have confirmed that the zein domain appended to the C-terminus of polypeptides with their own signal peptide for translocation in the ER, or an extended version (Zera^®^) that includes the zein signal peptide and can thus be placed at the N-terminus, can successfully trigger PB formation and high accumulation when fused to diverse recombinant proteins such as bean phaseolin (Mainieri et al., 2004), enhanced cyan fluorescent protein (Llop-Tous et al., 2010), xylanase (Llop-Tous et al., 2011), DsRed (Joseph et al., 2012), and human papillomavirus (Whitehead et al., 2014) and can be used not only in vegetative plant tissues but also in fungal, insect, and mammalian cells (Torrent et al., 2009a).

The accumulation, folding, and post-translational modification of recombinant proteins depend on their subcellular localization, because the cytosol, the ER, peroxisomes, and the different subcompartments of semiautonomous organelles have specific chemical features and repertoires of protein folding helpers. Targeting strategies must therefore be adapted for each candidate protein, and the addition of signal sequences to direct proteins to different subcellular compartments is often used to optimize production (Hofbauer and Stoger, 2013). Natural storage organelles originate from the endomembrane system and most ectopic PBs have accordingly been derived from the ER, whereas PB induction in other compartments has met with only limited success (Bellucci et al., 2005; De Marchis et al., 2012). Here, we used N-terminal portions of 27 kDa γ-zein as fusion partners for the visual marker DsRed, and the fusion proteins were directed to different compartments to investigate whether PBs can be encouraged to form outside the endomembrane system. We found that constructs with a plastid-targeting peptide led to the induction of PBs, but that constructs lacking this peptide were nevertheless able to form PBs associated with plastids, revealing the presence of a cryptic plastid-targeting signal within the PB-inducing N-terminal region of the mature γ-zein. We provide confocal and electron microscopy evidence for the localization of the PBs in different compartments within the plastid and consider the nature of the plastid import pathways.

## Materials and Methods

### Vector constructs

**Figure 1:**
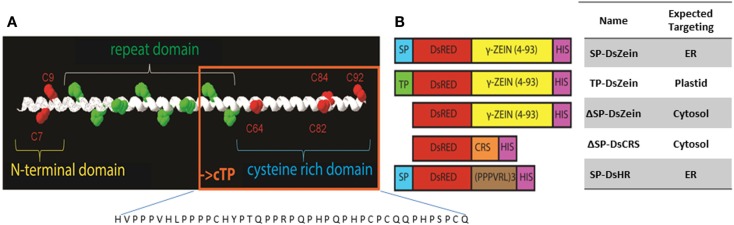
**(A)** The N-terminal sequence of mature 27 kDa γ-zein, which has been shown to trigger PB formation. The sequence comprises two N-terminal cysteine residues (red) followed by an amphipathic repeat region and another four cysteine residues. The sequence present in construct ΔSP-DsCRS is boxed in orange. **(B)** Schematic overview of the expression constructs. SP, signal peptide for entry into the ER; TP, plastid-targeting peptide; DsZein, fluorescent marker protein plus γ-zein(4–93); CRS, cysteine-rich sequence (residues 51–93); HR, hydrophobic repeat sequence (PPPVRL)_3_; HIS, polyhistidine tag.

All cloning steps were carried out with the binary vector pTRA, a derivative of pPAM (GenBank AY027531). Tetrameric DsRed (Jach et al., 2001) was joined via a (GGGS)_2_ linker to residues 4–93 of the mature 27 kDa γ-zein protein (lacking the signal peptide) in the same way as described for the PB-forming phaseolin fusion construct zeolin (Mainieri et al., 2004) to generate our basic vector ΔSP-DsZein. In construct SP-DsZein, a plant codon-optimized signal peptide sequence derived from a murine antibody (SP) was added at the N-terminus to direct the protein into the secretory pathway. In construct TP-DsZein, the 69-amino-acid plastid transit peptide sequence from the barley GBSSI gene (accession no. AF486514) was added instead. A peptide analysis using ChloroP prediction program (http://www.cbs.dtu.dk/services/ChloroP/) identified a potential chloroplast targeting peptide of 43 residues (cTP score of 0.460) in the N-terminal part of the mature 27 kDa γ-zein protein. This sequence comprising residues 51–93 of the γ-zein sequence was fused to DsRed via the (GGGS)_2_ linker to test the predicted function of this peptide. The resulting construct was termed ΔSP-DsCRS. For the control construct SP-DsHR, we used three repeats of the sequence PPPVRL fused to DsRed. This sequence is similar to the repeat domain of the zein sequence and does not include cysteine residues. A His_6_ tag was added to the C-terminus of all constructs as an additional means for detection. The synthetic fusion sequences were inserted into the pTRA-vector between the *Tobacco etch virus* (TEV) 5′-untranslated region and the *Cauliflower mosaic virus* (CaMV) 35S terminator (Figure 1). The expression construct was thus placed under the control of the CaMV 35S promoter with duplicated transcriptional enhancer.

### Plant material

Tobacco (*Nicotiana tabacum* cv. SR1) plants were cultivated in soil in growth chambers at 26°C and 70% humidity with a 16-h photoperiod. The TOC-GFP tobacco marker line, expressing a marker that highlights the outer plastid envelope, was kindly provided by Dr. Maureen Hanson, Cornell University, Ithaca, NY, USA (Hanson and Sattarzadeh, 2008) and cultivated under the same conditions.

### Agroinfiltration of tobacco leaves

*Agrobacterium tumefaciens* (GV3101) preserved as a glycerol stock was inoculated into 5 ml aliquots of YEB medium containing 25 mg/l kanamycin, 25 mg/l rifampicillin, and 50 mg/l carbenicillin, and incubated for 2 days at 28°C, shaking at 180 rpm. The OD_600_ of the culture was adjusted to ~1.0 with 2× infiltration medium (100 g/l sucrose, 3.6 g/l glucose, 8.6 g/l MS salts, pH 5.6) and 200 μM acetosyringone was added before infiltration into tobacco leaves. The infiltrated leaves were harvested 3–10 days post-infiltration (DPI).

### Protein immunoblot analysis

Infiltrated leaves (7 DPI) were harvested and ground in liquid nitrogen to a fine powder. For each sample, 60 mg leaf powder were extracted in 200 μl phosphate buffered saline (PBS, pH 7.4) and shaken for 15 min at room temperature. After centrifugation at 13,000 × *g* for 15 min at room temperature, the supernatant (PBS-soluble fraction) was transferred to a fresh tube and the pellet (insoluble fraction) was washed three times with PBS before re-extraction with buffer K (62.5 mM Tris pH 7.4, 10% glycine, 5% 2-mercaptoethanol, 2% SDS, 8 M urea). The relative quantity of total recombinant protein was estimated by directly homogenizing 60 mg leaf samples in 200 μl buffer K, sonicating for 20 s, and centrifuging as above for 10 s. Protein samples were boiled in loading buffer, separated by SDS-PAGE in 12% acrylamide gels and transferred to a nitrocellulose membrane. DsRed-fusion proteins were detected using an anti-His-tag antibody. The recombinant protein was visualized with an alkaline phosphatase-conjugated goat-anti-mouse IgG (Promega, Fitchburg, WI, USA) diluted 1:5000. Images were analyzed using Image Lab v5.1 (Bio-Rad Laboratories, Hercules, CA, USA).

### Fluorescence and electron microscopy

Infiltrated leaves were cut into small pieces with a razor blade. For fluorescence microscopy, the samples were mounted in tap water on a glass slide and DsRed fluorescence was observed under a Leica SP5 confocal laser scanning microscope (CLSM).

For immunolocalization by electron microscopy, the samples were fixed in 4% (w/v) paraformaldehyde plus 0.5% (v/v) glutaraldehyde in 0.1 M phosphate buffer (pH 7.4) at 4°C overnight, then dehydrated through an ethanol series and polymerized in LR White resin as previously described (Arcalis et al., 2010). Ultra-thin sections were mounted on gold grids, blocked with 5% (w/v) bovine serum albumin in 0.1 M phosphate buffer (pH 7.4) and incubated with the polyclonal Living Colors DsRed antibody. The samples were then incubated with a rabbit-anti-goat IgG linked to 10-nm colloidal gold and stained in 2% aqueous uranyl acetate.

For ultrastructural studies by electron microscopy, the samples were fixed in and in 2% (w/v) paraformaldehyde plus 2.5% (v/v) glutaraldehyde in 0.1 M phosphate buffer (pH 7.4) at 4°C overnight followed by an additional post-fixing step where samples were incubated in 1% (w/v) osmium tetroxide with 0.8% (w/v) KFeCN in 0.1 M phosphate buffer (pH 7.4) for 3 h at room temperature. After dehydration through an acetone series, the samples were polymerized in Spurr epoxy resin as previously described (Arcalis et al., 2010) and observed under a FEI Tecnai G^2^ transmission electron microscope.

## Results

### Protein bodies can form outside the ER and the compartment determines their morphology and the solubility of the stored recombinant protein

**Figure 2:**
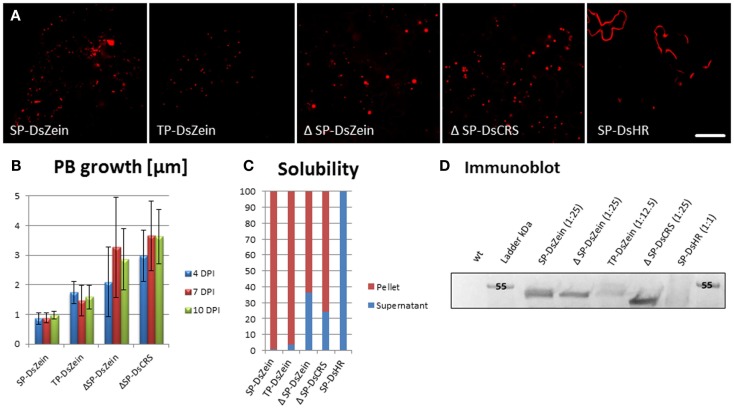
**(A)** Overview of the fluorescent signal distribution following transient expression with the constructs shown in Figure 1. **(B)** Protein body growth and size distribution after infiltration. Bars represent the standard error; *n* = 25. **(C)** Solubility of the recombinant fusion proteins. Leaf samples were extracted in PBS (soluble fraction) and pellets were then re-extracted under strong reducing conditions. **(D)** Immunoblot analysis of total protein extracts from leaf samples. Antiserum against the HIS-tag was used to detect the recombinant protein.

The N-terminal portion of γ-zein is necessary for the ability of this protein to form PBs in the ER (Geli et al., 1994). To investigate whether PBs can also be created in other compartments, we designed a fusion protein comprising DsRed and 89 residues of γ-zein starting from the fourth amino acid after the N-terminal signal peptide cleavage site, hereafter γ-zein(4–93). This is the same zein fragment that promotes the formation of ER-located PBs when fused at the C-terminus of the vacuolar storage protein phaseolin, in the chimeric protein zeolin (Mainieri et al., 2004). The fragment was the basis for different constructs targeting the fusion protein to the cytosol (ΔSP-DsZein), ER (SP-DsZein), and plastids (TP-DsZein). As control, we used a sequence that is similar to the hydrophobic domain in γ−zein(4–93) but lacks any cysteine residues, and thus lacks the ability to form PBs (SP-DsHR). An overview of these constructs is provided in Figure 1. PB formation was induced with all constructs except the negative control (Figure 2A). A comparison of PB biogenesis and growth in the different compartments indicated that PBs induced by the SP-DsZein and TP-DsZein constructs were limited to a size of 1–1.5 μm, which was achieved ~4 DPI, and showed a homogeneous size distribution. In contrast, the PBs induced by ΔSP-DsZein had an average diameter of ~3 μm and were heterogeneous in terms of size and growth rate (Figure 2B).

Infiltrated leaves were harvested 7 DPI and the solubility of the four DsRed-fusion proteins was determined by extraction in PBS and re-extraction of the pellet under strong reducing conditions. Following infiltration with SP-DsZein and TP-DsZein, most of the DsRed-fusion protein was only soluble after re-extraction. However, infiltration with ΔSP-DsZein yielded a minor soluble fraction, and following infiltration with the control construct SP-DsHR all of the recombinant fusion protein was soluble in PBS, as expected (Figure 2C).

The infiltrated leaves were also homogenized directly in strong reducing buffer. The total leaf extracts were then analyzed by immunoblot to confirm the molecular masses of the proteins and to compare the relative amounts of recombinant fusion protein obtained with the different constructs (Figure 2D). All PB-inducing fusion proteins led to higher yields of the chimeric protein than the control construct for secretion, SP-DsHR (Figure 2D). A further control lacking both the signal peptide and the cysteine-rich sequence, ΔSP-DsHR, resulted in even lower expression in *N. tabacum* and was not detectable by immunoblot or fluorescence microscopy (data not shown). Infiltration of this construct into *N. benthamiana* led to slightly higher expression levels and microscopy analysis confirmed that this construct caused cytosolic distribution of the fusion protein without induction of PBs (Figure S1 in Supplementary Material).

### SP-DsZein and TP-DsZein induce protein bodies in the ER and plastids, respectively

**Figure 3:**
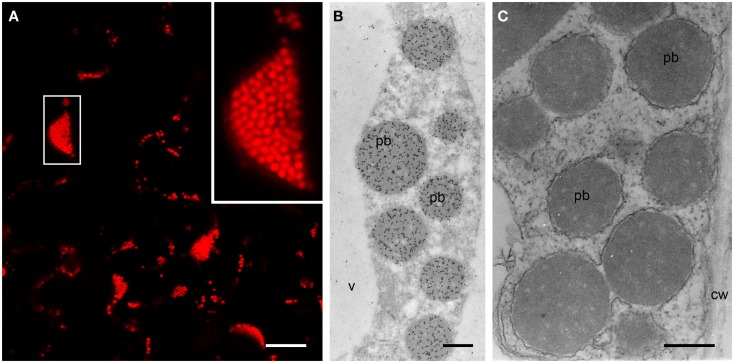
**Protein body formation in the ER (SP)**. **(A)** Confocal laser scanning microscopy. Infiltrated leaf tissue overview, showing abundant protein bodies. The inset shows an enlargement of the outlined protein body cluster. **(B,C)** Electron microscopy. Spherical protein bodies (pb) showing abundant, specific labeling for DsRed **(B)**, surrounded by a ribosome-studded membrane [**(C)**, arrowhead]. Cell wall (cw), vacuole (v). **(C)** Lipid staining. Bars = 25 μm **(A)** or 0.5 μm **(B,C)**.

**Figure 4:**
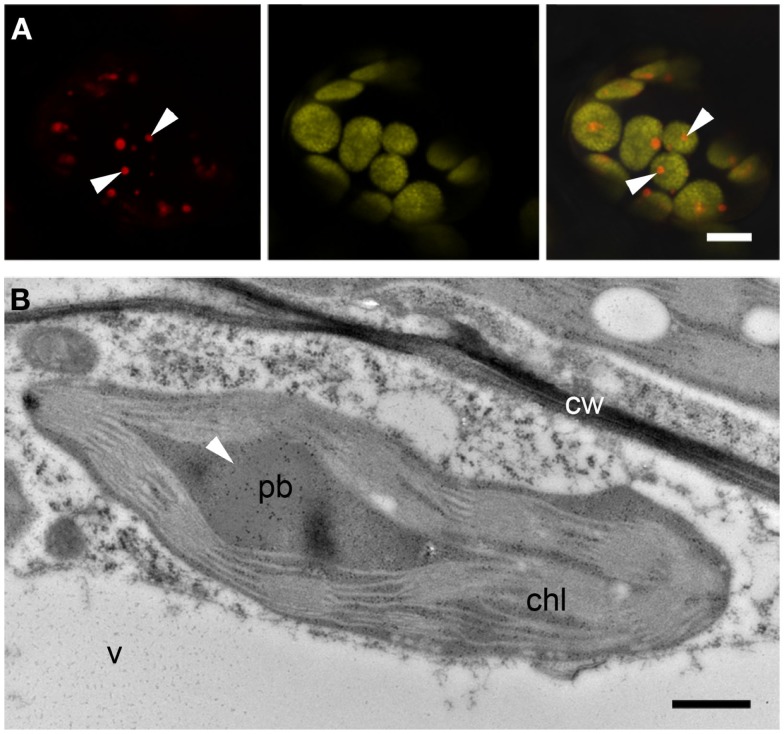
**Protein body formation in the plastids (TP)**. **(A)** Confocal laser scanning microscopy. Protein bodies (arrowheads) within the plastids. Left: DsRed fluorescence, middle: plastid autofluorescence, right: merged channels. Note that in most cases one protein body can be observed per plastid. **(B)** Immunoelectron microscopy. Protein body (pb, arrowhead) within the stroma of the plastid (chl). Mitochondria (m). Bars = 5 μm **(A)** or 0.5 μm **(B)**.

The formation of PBs was investigated by confocal microscopy 4–15 DPI. Construct SP-DsZein induced the formation of spherical bodies with a tendency to form clusters (Figure 3A) and electron microscopy confirmed the presence of PBs in the cytoplasm (Figure 3B), surrounded by a ribosome-studded membrane, indicating they had originated from the ER (Figure 3C). Construct TP-DsZein also induced the formation of spherical PBs but their distribution differed significantly from those induced by SP-DsZein. Confocal microscopy clearly revealed the formation of fluorescent PBs in the stroma (Figure 4). Most plastids contained a single PB in the stroma (Figure 4A). PBs formed by SP-DsZein are, like natural PBs of maize endosperm, in close contact to the inner face of the ER membrane (Figure 3B). Those formed by TP-DsZein are only in part, but not completely, in contact with thylakoids.

### The ΔSP-DsZein construct induces protein bodies associated with the plastid envelope

**Figure 5:**
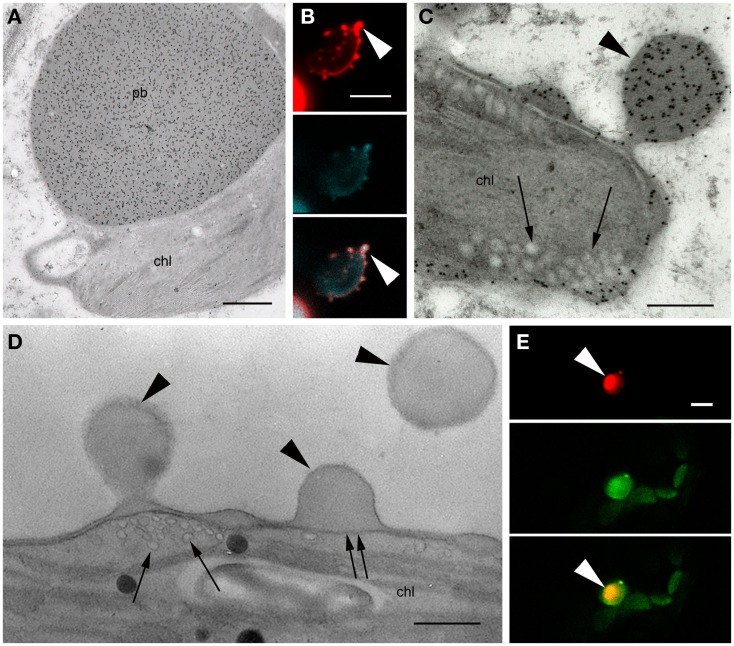
**Protein bodies induced by ΔSP-DsZein**. **(A)** Immunoelectron microscopy. Gold particles decorating a protein body (pb) in close association with a plastid (chl). **(B)** CLSM image showing the accumulation of fluorescent fusion protein in the periphery of a plastid, as well as several budding sites (arrowhead). DsRed fluorescence (top), autofluorescence of plastids (middle), and merged channels (bottom) are shown. **(C)** Immunoelectron microscopy, localization of DsRed. Abundant gold probes are visible in the periphery of a plastid, showing a budding protein body (arrowhead). Note the tubular thylakoids in the vicinity of the budding site (arrows). **(D)** Detection of unsaturated lipids, by electron microscopy. Budding protein bodies are confined by the outer membrane (arrowheads) and the inner envelope membrane (double arrow) of the plastid. Tubular thylakoids can be observed at the budding site (arrows). **(E)** CLSM image showing red fluorescent protein bodies enclosed by the outer plastid envelope membrane highlighted with a TOC-GFP membrane marker (arrowheads). DsRED (top), GFP (middle) and merged channels (bottom) are shown. Bars = 0.5 μm **(A,D)**, 5 μm **(B,E)**, or 0.25 μm **(C)**.

The transient expression of a construct lacking an N-terminal signal sequence (ΔSP-DsZein) induced the formation of large PBs (~3 μm in diameter) that appeared to be localized mostly in close association with the plastids (Figure 5A). Some plastids were characterized by an irregular red fluorescent periphery, suggesting that smaller PBs were budding from the plastid membrane (Figure 5B). Indeed, electron microscopy revealed several PBs budding from the plastid surface (Figures 5C,D). Interestingly, tubular thylakoids [typically a sign of plastid stress; (Monselise et al., 1984)] were observed in the vicinity of these budding sites (Figures 5C,D). Detailed images revealed that recombinant protein accumulating at the plastid periphery was actually confined by two membranes, one enclosing the PB on the outside and the other defining the stromal border, indicating that the recombinant protein is localized in the intermembrane space (IMS), between the inner and outer membranes of the plastid envelope (Figure 5D). On several instances the PB protrudes from the chloroplast, suggesting a process of budding from the intermembrane space (Figures 5C,D). Lipid staining revealed that the budding PBs are enclosed in a lipid membrane derived from the outer membrane of the plastid envelope (Figure 5D).

To confirm these results, the ΔSP-DsZein construct was transiently expressed in the leaves of a TOC-GFP tobacco marker line, which expresses a fluorescent marker that identifies the outer plastid membrane (Hanson and Sattarzadeh, 2008). CLSM allowed the collection of z-series images from plastids and associated red fluorescent PBs, confirming that the PBs were covered by the outer plastid membrane (Figure 5E).

### The cysteine-rich sequence of γ-zein contains a cryptic plastid-targeting signal

To narrow down the region in the N-terminal portion of γ-zein responsible for the *de novo* formation of PBs in the plastid envelope, we designed a further construct containing DsRed and the distal part of the γ-zein(4–93) fragment described above, hereafter γ-zein(51–93). The construct was named ΔSP-DsCRS because the short zein fragment retains the four Cys residues that follow the repeated region and is therefore a cysteine-rich sequence (CRS).

**Figure 6:**
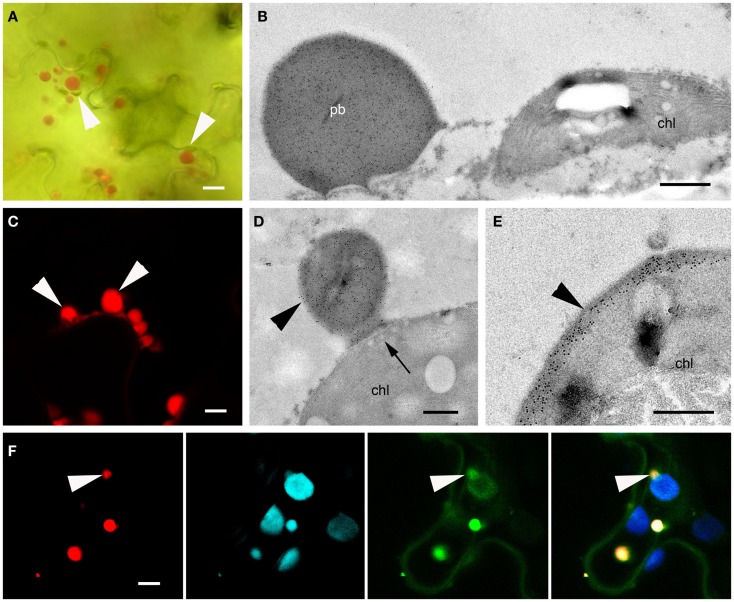
**Protein bodies induced by ΔSP-DsCRS**. **(A)** Bright field microscopy. Several large fluorescent protein bodies can be observed, some in close association with a plastid (arrowhead). **(B)** Immunoelectron microscopy, localization of DsRed. Large protein body in the vicinity of a plastid (chl). **(C)** CLSM image of protein bodies budding off a plastid (arrowheads). **(D,E)** Immunoelectron microscopy, localization of the fluorescent fusion protein in the IMS. Protein body budding off a plastid [**(D)**, arrowhead, chl], showing tubular thylakoids close to the budding site [**(D)**, arrow]. Abundant gold probes in the periphery of the plastid [**(E)**, arrowhead]. **(F)** CLSM image of red fluorescent protein bodies enclosed by the outer plastid envelope membrane highlighted with a TOC-GFP membrane marker (arrowheads). DsRed (far left), plastid autofluorescence (middle-left), GFP (middle-right), and merged channels (far right) are shown. Bars = 10 μm **(A)**, 0.5 μm **(B,D–F)**, or 5 μm **(C)**.

This construct behaved similarly to ΔSP-DsZein, forming large and heterogeneous PBs that grew at different rates and in some cases reached up to 5 μm in diameter (Figures 2A,B and 6A–C). As with ΔSP-DsZein, a relevant fraction of the DsRed-fusion protein was soluble in the absence of reducing agent (Figure 2C). By 15 DPI, large and irregular PBs were observed, which appeared to be budding from the plastid surface (Figures 6C,D). These large fluorescent PBs could even be observed by bright field microscopy (Figure 6A). Electron microscopy revealed frequent associations between these PBs and the plastids, as described for ΔSP-DsZein, and smaller PBs could again be observed budding from the plastid envelope (Figure 6D). Occasionally, the fusion protein is distributed as electron dense material within the intermembrane space without forming a spherical PB (Figure 6E). Transient expression in the leaves of TOC-GFP tobacco plants again confirmed that the PBs were covered by the outer plastid membrane (Figure 6F).

## Discussion

### PB formation in the chloroplast stroma

Protein bodies have been induced mainly as derivatives of the endomembrane system, whereas there have been few attempts to induce PBs in the cytosol or plastids (Bellucci et al., 2005, 2007; De Marchis et al., 2011). We used DsRed fused to different stretches of the N-terminal portion of mature γ-zein and designed constructs targeting the cytosol, ER, and plastids. All three constructs were able to induce PBs, however, a control construct containing an amphipathic repeat domain similar to the zein sequence but lacking the cysteine-rich element was unable to form PBs, as expected from previous mutagenesis experiments performed on γ-zein or fusions derived from it (Pompa and Vitale, 2006).

It was somewhat surprising that the TP-DsZein construct was able to induce the formation of DsRed PBs within the plastids, whereas the phaseolin-zein fusion protein zeolin was unable to form PBs in the plastid and instead accumulated as monomers without disulfide bonds (Bellucci et al., 2007; De Marchis et al., 2011). This discrepancy may reflect the different expression strategies – zeolin was produced by plastid transformation followed by direct accumulation within the plastid, whereas TP-DsZein was produced by transient expression in the nucleus followed by targeting to the plastid as a post-translational event. Alternatively, the different intrinsic properties of the proteins may be relevant – DsRed is a well-established marker which is known to accumulate in plastids whereas phaseolin might interfere with protein homeostasis in the plastid or may not reach the minimum concentration necessary for PB formation (Gutierrez et al., 2013). Several post-translational modifications are known to take place in the stroma of tobacco plastids (Glenz et al., 2006) including the formation of disulfide bonds (Bally et al., 2008). A number of previous reports describe the expression, in the plastid stroma, of biologically functional proteins that require intra-chain and/or inter-chain disulfide bonds for their activity (Staub et al., 2000; Daniell et al., 2001; Lakshmi et al., 2013).

### PB formation in the intermembrane space

The basal construct lacking an N-terminal signal peptide (ΔSP-DsZein) resulted in the unexpected formation of PBs associated with the plastid periphery. This distribution was clearly distinct from the stromal localization mediated by TP-DsZein, and electron microscopy duly confirmed that the protein was accumulating not in the stroma, but in the IMS of the plastid envelope. This was confirmed by transient expression in transgenic tobacco plants expressing a fluorescent marker protein highlighting the outer plastid envelope. The ectopic PBs containing DsRed were budding off from the envelope into the cytoplasm. Whereas SP-DsZein and TP-DsZein induced the formation of homogeneous PBs that were insoluble under non-reducing conditions, the PBs formed by ΔSP-DsZein varied considerably in size and shape, and a significant proportion of the recombinant protein was soluble under non-reducing conditions indicating that it was not assembled into a disulfide-bonded polymer. This may indicate that targeting of the protein to the plastids was only partial. Indeed, it was not clear whether all the observed PBs had originated by budding from chloroplasts and we therefore cannot exclude the possibility that further PB-like fluorescent structures had also formed directly in the cytosol. Alternatively, the partial recovery of DsRed by extraction with a non-reducing buffer could mean that part of the protein that enters the IMS fails to form insoluble polymers. The finding that the solubility of γ-zein in non-reducing conditions is inversely related to the number of Cys residues in the N-terminal region supports this assumption (Mainieri et al., 2014). Whereas redox processes in the stroma and thylakoid lumen are well characterized, the redox state of the IMS has not been studied in detail (Herrmann et al., 2009; Stengel et al., 2010). Redox machineries equivalent to those catalyzing the oxidation of proteins in the periplasmic space of bacteria or the IMS of mitochondria have not been identified in the IMS of plastids, although it is a topologically equivalent compartment. It has been suggested, however, that TOC12, a subunit of the translocon on the outer chloroplast membrane (TOC) complex in the plastid IMS, may be oxidized as part of the redox-dependent regulation of plastid protein import (Balsera et al., 2010). Once it has crossed the outer plastid membrane the DsZein fusion protein may therefore assemble into disulfide-linked, insoluble polymers that are no longer competent for further transport.

### A plastid-targeting signal in γ-zein

It is important to note that in the absence of the added γ-zein(4–93) sequence the DsRed sequence we used did not accumulate in tobacco plastids and did not form PBs (Jach et al., 2001). This suggests that the N-terminal γ-zein sequence is responsible for plastid targeting when fused to the C-terminus of DsRed. Chloroplast transit peptides are usually located at the N-terminus and are removed upon protein translocation, however, imported proteins exist that do not have removed peptides and contain targeting information at different locations along the mature sequence (Miras et al., 2002). We therefore sought the core sequence responsible for conferring this property, i.e., a cryptic plastid-targeting signal within the γ-zein sequence. Plastid transit peptides usually contain a high proportion of hydrophobic and basic amino acids but few acidic residues (Zhang and Glaser, 2002). The γ-zein CRS extending from residues 51–93 shares these properties and appeared to be the most likely plastid-targeting sequence based on *in silico* analysis (Emanuelsson et al., 2000). Indeed, when this sequence was fused to DsRed lacking any further targeting information (construct ΔSP-CRS) we observed plastid targeting and the formation of fluorescent PBs in the IMS, just as we observed with the complete γ-zein N-terminal sequence, indicating that the CRS is the relevant sequence part causing plastid targeting. It is possible, however, that unidentified sequence motifs in the DsRed polypeptide may additionally contribute to targeting of the fusion.

**Figure 7:**
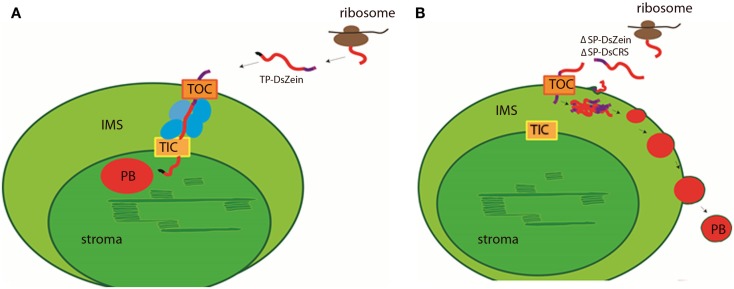
**Hypothetical model for the import of the fluorescent fusion proteins and for PB formation in the plastid**. **(A)** TP-DsZein likely enters the plastid via the TOC/TIC pathway (orange). After passing the TOC complex, it is probably recognized by the IMS translocation complex (blue) and further transported via the TIC channel into the stroma where the transit peptide (black) is removed and a PB (red) is formed. **(B)** ΔSP-DsZein and ΔSP-DsCRS may enter the plastid via the TOC complex or a non-canonical route. After crossing the outer plastid membrane the fusion protein assembles into insoluble polymers that are no longer competent for further transport. PBs are formed in the IMS and bud off from the plastid. The DsRed sequence is shown in red, γ-zein sequences are shown in purple, ribosomes are marked in brown.

It is unclear whether the ΔSP-DsZein protein enters the plastid via the TOC/TIC complexes (Li and Chiu, 2010) or a non-canonical route (Armbruster et al., 2009). The TOC/TIC pathway involves hetero-oligomeric complexes in the inner and outer plastid membranes (Jarvis and Soll, 2002; Kessler and Schnell, 2009). Import via the TOC channel requires at least four different TOC proteins, all sharing conserved cysteine residues (Stengel et al., 2009). The formation and reduction of disulfide bonds between TOC proteins was shown to regulate this trafficking step (Kessler and Schnell, 2009), but there is no evidence for the formation of disulfide bonds between TOC components and incoming pre-proteins (Stengel et al., 2010). However, the cysteine residues in the γ-zein CRS are probably exposed and in a reduced form in the cytosol, so the formation of disulfide bridges with TOC components might also occur and prevent further transport through the TIC receptor. Instead, the protein would remain in the IMS, where apparently the conditions are appropriate to support the formation of PBs. In contrast, TP-DsZein is probably recognized by the IMS translocation complex and therefore further transported via the TIC channel (Figure 7). Interaction studies will be necessary to characterize the import pathways in more detail. It was not possible to regenerate stable transgenic plants transformed with the ΔSP-DsZein and TP-DsZein constructs (data not shown) suggesting that the formation of PBs in the plastids was not compatible with the regeneration of viable plants, perhaps because the PBs disturbed the sensitive redox equilibrium in the plastids (Wittenberg and Danon, 2008).

It was remarkable that PBs not only formed in the plastid IMS but also budded from the surface in a similar manner to the normal budding of prolamin bodies from the ER in cereal endosperm cells. This indicates that both the biogenesis and the budding of PBs are independent of ER-specific factors, providing insight into the formation of endogenous storage organelles in cereal seeds. Although the mechanism of PB biogenesis is not fully understood, two driving forces have been previously identified and attributed to the γ-zein N-terminal sequence, i.e., the formation of inter-chain disulfide bonds between cysteine residues and lateral interactions involving the amphipathic repeats (Llop-Tous et al., 2010). Both elements are considered necessary, so it is perhaps surprising that the CRS (which includes four cysteine residues but only a small part of the amphipathic repeat region) was sufficient to induce PB formation. This might be attributed to the design of our fusion construct, where tetrameric DsRed forms the N-terminal part and may support oligomerization, thus, functionally replacing the amphipathic repeats. The fusion partner has also been shown to influence PB biogenesis in previous studies. For example, phaseolin is a soluble homotrimer (Vitale et al., 1995) and induces efficient zeolin PB formation when fused to the γ-zein N-terminal sequence (Mainieri et al., 2004). In contrast, PBs were not formed when the *Human immunodeficiency virus* Nef antigen was fused to the γ-zein N-terminal sequence, whereas PB formation was possible when Nef was fused to the entire chimeric protein zeolin (De Virgilio et al., 2008).

## Concluding Remarks

We have shown that PBs can be induced to form outside the endomembrane system, and we have identified a cryptic plastid-targeting signal in the N-terminal portion of the 27 kDa γ-zein protein. We have shown that this cysteine-rich sequence mediates the transport of DsRed across the outer plastid envelope and can induce the formation of PBs in the plastid IMS. These results add to our knowledge on endogenous and ectopic PB formation by demonstrating that PB formation and budding do not require ER-specific factors. It will be interesting to attempt the formation of plastid-targeted PBs with other fusion proteins to determine the robustness of this strategy for recombinant protein production in transient expression systems.

## Conflict of Interest Statement

The authors declare that the research was conducted in the absence of any commercial or financial relationships that could be construed as a potential conflict of interest.

## Supplementary Material

The Supplementary Material for this article can be found online at http://www.frontiersin.org/Journal/10.3389/fbioe.2014.00067/abstract
