# Supplementary figures and images for: The Induction of Recombinant Protein Bodies in Different Subcellular Compartments Reveals a Cryptic Plastid-Targeting Signal in the 27-kDa γ-Zein Sequence
Source: Front Bioeng Biotechnol. 2014 Dec 11;2:67. doi: 10.3389/fbioe.2014.00067 (PMC4263181; doi:10.3389/fbioe.2014.00067)

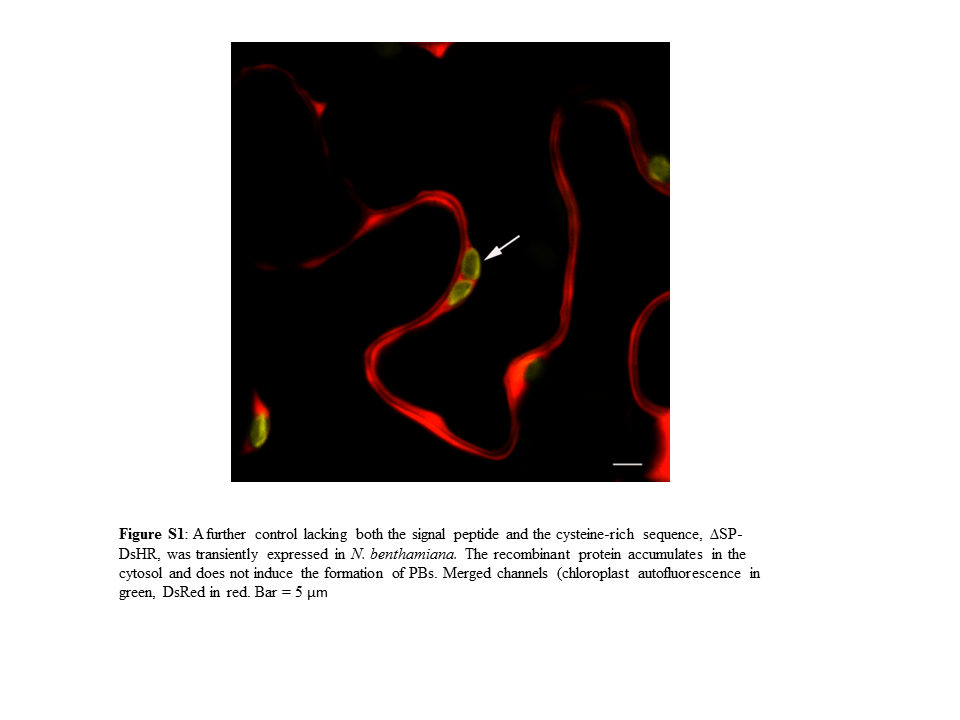

Supplement: Supplementary file 1 [file Image_1.TIF]
